# Who is sexually active? Using a multi-component sexual activity profile (MSAP) to explore, identify and describe sexually-active high-school students in rural KwaZulu-Natal, South Africa

**DOI:** 10.1186/s12889-019-6602-y

**Published:** 2019-03-18

**Authors:** Hilton Humphries, Farzana Osman, Lucia Knight, Quarraisha Abdool Karim

**Affiliations:** 1 Centre for the AIDS Programme of Research in South Africa (CAPRISA), Nelson R Mandela School of Medicine, University of KwaZulu-Natal, Durban, South Africa; 2 School of Public Health, University of the Western Cape, Bellville, South Africa; 3 Department of Epidemiology, Mailman School of Public Health, Columbia University, New York City, USA

**Keywords:** Sexual activity, Risk profiles, Sexual reproductive health, HIV, Young people, School students

## Abstract

**Background:**

Understanding sexual activity is necessary to prevent sexually transmitted infections. Evidence from Sub-Saharan Africa suggests that 10–20% of youth aged 15–24 are sexually active before reaching 15 years, yet estimating sexual activity remains challenging. This study explored the use of multiple sexual health outcomes to identify sexually-active young women in rural KwaZulu-Natal, South Africa.

**Methods:**

Using a multi-component sexual activity profile (MSAP), we aimed to identify sexually active students. Based on data from 2675 grade 9 and 10 students attending 14 high schools) in rural KwaZulu-Natal, we constructed a descriptive diagram identifying students who were sexually active by self-report vs MSAP profile. T-tests for two independent samples was performed to compare by sex and ecological variables that characterise students newly-identified as sexually active.

**Results:**

Using self-report only, 40.3% self-reported as sexually active, whilst the MSAP identified 48.7% (223 additional students). More females were identified than males. Younger adolescents were more likely to underreport sexual activity but were identified using MSAP. Newly-identified as sexually active were more likely to be female (*p* = < 0.000), 15 years old or younger (*p* = 0.008), less likely to perceive being at risk (*p* = 0.037) or have ever used alcohol (*p* = < 0.000). At a relational level, they were less likely to report having ever had a boyfriend/girlfriend (*p* = 0.000) or to have felt pressured to have sex by their peers (*p* = < 0.000) or partners (*p* = 0.008). At a familial level they more likely to be of medium socioeconomic (SES) status (*p* = 0.037) whilst at a school and community level they were less likely to have repeated a grade (*p* = 0.024) and were more likely to be engaged in social activities (*p* = 0.032).

**Conclusions:**

The MSAP profile identified more potentially sexually active students, and gave insight into the characteristics of students who may be unwilling to self-report sexual activity Future work should investigate how this approach could enhance and describe sexually-active adolescents for research and healthcare provision.

## Background

Unprotected sexual encounters are the key cause of sexually transmitted infections. Evidence from Sub-Saharan Africa (SSA) suggests that 10–20% of young people aged 15–24-years-old are sexually active before the age of 15 years [1]. Accurately estimating sexual activity in young people remains challenging, self-report measures tend to underreport sexual activity due to social desirability bias, experimenter effects, and a reluctance to admit early sexual engagement [2–4]. However, identifying sexually active adolescents is critical, considering sexually transmitted infections (STIs) account for a major health burden in this group [1, 5, 6]. Globally, over 40 million young people are infected with herpes simplex virus (HSV-2) [7] and four million infected with HIV [6]. Young women in SSA remain the most vulnerable group [6, 8, 9]; studies from South Africa indicate school-going women aged 13–24-years-old already have a 6% HIV prevalence, 10.7% HSV-2 prevalence and 3.6% pregnancy prevalence [10].

Sexual activity is an important and widely used proxy indicator for increased risk of possible HIV, STI infection or pregnancy, especially considering the low levels of condom use amongst young people [9, 11]. Research on obtaining better approximations of sexual activity is limited [12]. The use of multiple data-points to get better estimations on sensitive topics has been used in the field of adolescent pregnancy. With similar limitations to self-reported sexual activity data, researchers in the field of adolescent pregnancy have had to use multi-data variable approaches to improve their estimates [3, 13]. The researchers used multiple data-point profiles which include reported sexual behaviour, contraceptive coverage from large surveys, and abortion rates in combination with adolescent birth rates to obtain better estimates of actual adolescent pregnancy rates [13, 14]. A similar approach of combining several data points could assist in identifying sexually active adolescents.

Using self-reported sexual activity, as well as other markers of possible sexual activity (HIV, HSV2, pregnancy, STI symptoms and previous pregnancies) could improve the estimation of sexual activity, and in particular unprotected sexual encounters. By better estimating sexual activity, comparisons between adolescents who, despite living in similar high-risk settings, are sexually active but never experience a negative health outcome, with those that do could be completed. This has the potential to provide the basis for additional analyses into how individual [9, 11, 15, 16], school [11, 17–19], partner and peer [20–22], familial [23–27], and the broader cultural and geographic level factors [20, 21, 28] affect risk outcomes. More accurate estimates of sexual activity could also assist in assessing whether adolescents correctly understand their risk of negative health outcomes, whether they underestimate their risk relative to their actual risk [29] and provide a basis for discussions about how they define being sexually active. Furthermore, the importance of increasing adolescent involvement in clinical trials means that better indicators of sexual activity and high-risk adolescents are essential.

This paper uses baseline data from a large school-based cluster randomised control trial to describe how a multi-component sexual activity profile (MSAP) can be used to estimate the proportion of sexually-active young people. Additionally, it aimed to understand the differences between those self-reporting sexual activity and those newly-identified using MSAP.

## Methods

### Study setting and population

The analysis uses baseline data collected during a cluster-randomised control trial (CAPRISA 007) that was conducted in a rural part of the uMgungundlovu district of KwaZulu-Natal, South Africa. A secondary data analysis from students attending 14 secondary schools in the district was included in this analysis. Data were analysed from all students in grades 9 and 10 who successfully enrolled. Details of the cohort selection and inclusion criteria are described elsewhere [10, 30].

### Study design and methods

The CAPRISA 007 study was a two-arm, matched pair, cash incentivised cluster randomised controlled trial that took place in the study schools between 2010 and 2013. The study has been presented and explained in more detail elsewhere [10, 30].

All participants provided informed consent prior to being enrolled. Students ≥18 years provided first-person consent following a literacy and comprehension assessment, whilst students < 18 years, provided assent, while consent was obtained from the parent/guardian. In the event that a parent/guardian was not available, proxy parental consent was obtained from a member of the School Research Support Group (SRSG). Behavioural and demographic data were collected using self-report, privately self-completed, structured questionnaires available in *isiZulu* and English. Biological measures included students’ HIV test results, HSV-2 results, and urine pregnancy test result. The details of the measures used in the parent study are discussed elsewhere [10]. All participants received referrals as required. All students consented that their data may be used in additional analyses. This study explores how we estimate sexual activity, and students are not personally identified about incongruencies in reported sexual activity. All ethical approvals were granted by the University of KwaZulu-Natal Biomedical Ethics Committee (BF105/010 and BE 523/14).

### Measures

We aimed to understand how many students were sexually active using self-reported sexual activity versus a combination of variables including biological and self-reported behavioural markers to create a Multicomponent Sexual Activity Profile (MSAP). For the purposes of this study, self-reported sexual activity was defined as any learner who reported to have ever had vaginal, anal or oral sex. For the MSAP, we included biological variables including: HIV test results, HSV-2 test results, pregnancy test results. MSAP also include self-reported behavioural variables: reported STI symptoms (those symptomatic screening questions used in the South African public health care system, including asking about experience of a vaginal/penile/urethral discharge, a genital sore or ulcer or pain on urination), previous pregnancy, and those who had made someone pregnant before. MSAP variables all suggest possible unprotected previous sexual encounters. The purpose was to identify the maximum number of young people who may have had a sexual encounter, however, it is possible that some students were HIV positive by vertical transmission, other modes of transmission (i.e. injecting drug-use) or reported not sexually active if they had engaged in sex through force. We include HIV positive in the definition as an individual may still pose a risk for infecting others if not virally suppressed. The MSAP variables identified students who self-reported as not sexually active, but whose profile suggested possible sexual activity. The MSAP variables can be easily collected and measured within a research setting [31].

### Statistical analysis

**Fig. 1:**
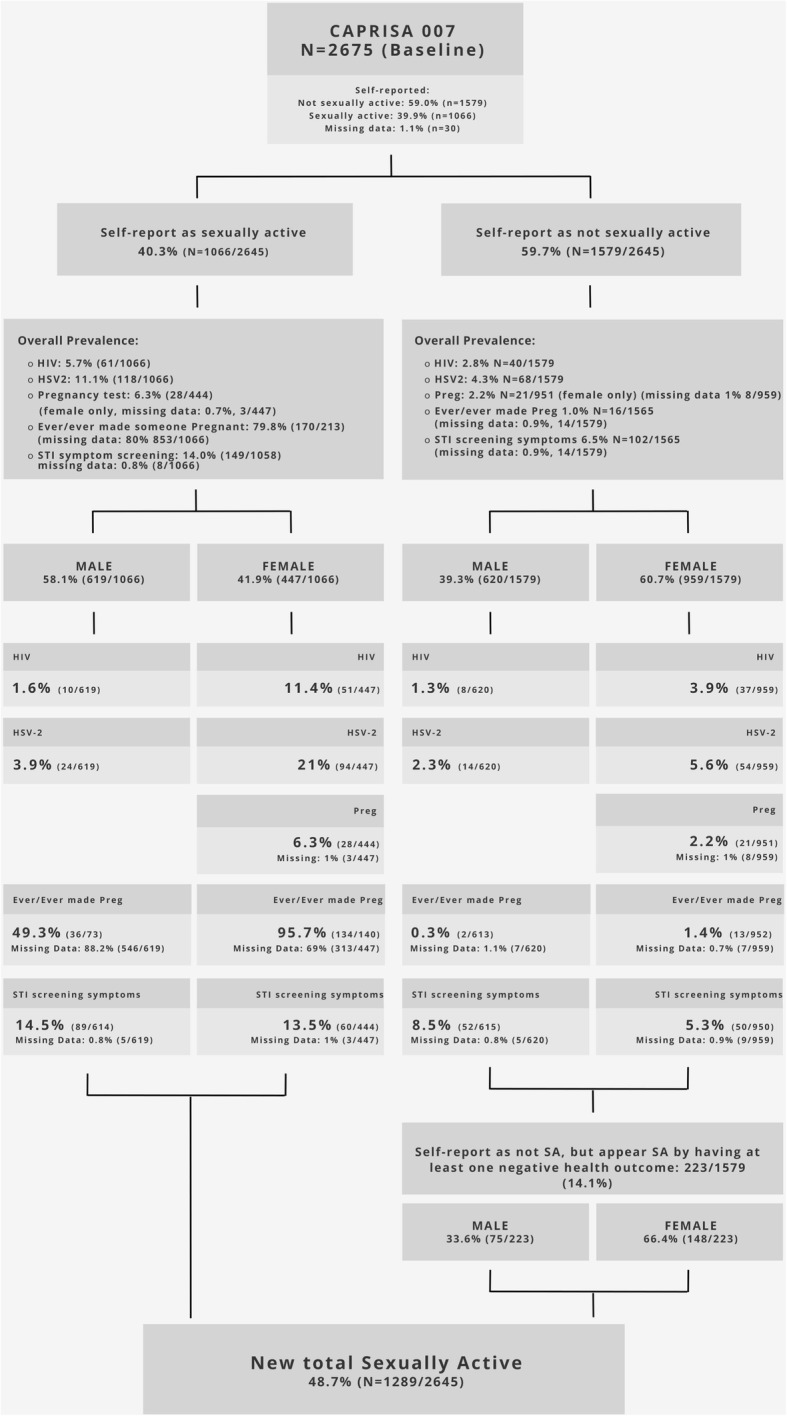
Flow diagram of biological and behavioural variables suggesting sexual activity in adolescent students self-identifying as either sexually active or not sexually active. # Note Percentage for ever pregnant/ever made someone pregnant in those reporting sexually active was calculated with regards to available data. Should be interpreted with caution as the denominator changed to 73 and 140 for male and females amongst those self-reporting as sexually active

Using a staged analysis of the data collected, we constructed a descriptive flow diagram that identified those students who disclosed as sexually-active and those that reported being not sexually active. In order to construct the flow diagram, several steps were followed to ensure we could identify the prevalence of key variables included in the MSAP (See Fig. 1).

The first step was to identify the individuals in the total cohort who had self-reported as either sexually active or not sexually active. The two groups were then split by sex as we expect to see variation in the MSAP by sex, with young women expected to bear a higher burden of each outcome. Descriptive summaries were generated to obtain the overall prevalence of each variable of the MSAP within each. HIV, HSV-2 and pregnancy prevalences were obtained using the outcomes of biological tests for each group, while prevalence data for experiencing an STI symptom and previous pregnancy/previously making someone pregnant were generated from self-report data collected through self-administered questionnaires. The prevalence of the biological markers (HIV, HSV-2, pregnancy) and the self-reported behavioural markers (previous/previously making someone pregnant and STI symptoms) were then calculated for each sub-group, reported as percentages. The prevalence of key MSAP variables within the group identifying as not sexually active was used to indicate sexual activity. The overall flow diagram was used to look at the difference of MSAP variables within each group, as well as highlighting differences between male and female students.

Summary statistics of the basic demographic data were generated for the overall cohort of students enrolled in the study as well as the groups identifying as sexually active or not. These variables were reported using medians for continuous variables and as frequency distributions for categorical data. As the data was collected from a cluster randomised control trial it was necessary to adjust for any cluster effects that may arise from the school-based sampling. The unadjusted analysis did not account for the clustering, calculating prevalence by combining prevalence of all schools (clusters). Using the adjusted prevalence estimates from each of the 14 clusters, a t-test for two independent samples was performed to compare by sex, reported sexual activity, and ecological variable [21, 32] differences between those newly identified vs self-reported as sexually active. As there were few clusters (< 20) in the main study, regression methods were not completed due to concerns over robustness [33].

## Results

The total grade 9 and 10 school student population in the 14 schools sampled was 3781. The analysis includes a total of 2675 (70.7%) students who provided consent or assent. Of these, 1423 (53.2%) were females and 1252 (46.8%) were males. The flow diagram of identifying the prevalence of variables included in the MSAP for those self-identifying as sexually active versus not sexually active is presented in Fig. 1. In total, 40.3% self-reported as being sexually active, whilst 59.7% of the students reported having never had any type of sex before. A total of 30 students did not complete the question on whether they were sexually active. These 30 students were negative for all variables constituting the MSAP and were excluded.

The basic demographics for the whole cohort, as well as the two groups with different self-reported sexually active status, are reported in Table 1. The overall median age of all students was 16 years (IQR 15–18), with a median age of 17 years (IQR 16–18) for males and 16 years (IQR 15–17) for females. The overall age range of students was 12–28 years for males, and 13–24 years for females. For those students who self-report as sexually active, the median age of students was 17 years (IQR 16–19) for males and 17 years (IQR 16–19) for females. The age range of self-reported sexually active students was 12–28 years for males, and 13–23 years for females. For those students who self-report as not sexually active, the median age of students was 16 years (IQR 15–17) for males and 15 years (IQR 15–16) for females. The age range of self-reported not sexually active students was 13–25 years for males, and 13–24 years for females. Overall, young men were more likely to report having been sexually active (50.0%) than their female counterparts (31.8%) (*p* = 0.001).

**Table 1:** Demographic and key MSAP characteristics for students in rural KwaZulu-Natal, South Africa

Sample Group	Variable		unAdj Male %	Adj%^a^	unAdj Female %	Adj% ^a^	Adjusted P
Overall (*N* = 2675)			*N* = 1252		*N* = 1423		
	Median Age		17 years (IQR 16–18)		16 years (IQR 15–17)	–	
	Age Range		12–28 years		13–24 years	–	
	Age	< 15	23.7 (297/1252)	25.6	41.5 (591/1423)	41.6	**0.002**
		16–17	40.3 (505/1252)	40.4	37.7 (537/1423)	37.5	0.383
		18–19	27.5 (344/1252)	26	14.9 (212/1423)	14.8	**0.005**
		> 20	8.5 (106/1252)	8.0	5.8 (83/1423)	6.1	0.518
	HIV Prev		1.4 (18/1252)	1.4	6.2 (88/1423)	6.4	**< 0.001**
	HSV-2 Prev		3.0 (38/1252)	2.6	10.4 (148/1423)	10.7	**< 0.001**
	Pregnancy Prev		Not applicable		3.5 (49/1412)	3.6	Not applicable
	Prev Reported Preg/made pregnant		3.1 (38/1225)	3.3	10.5 (147/1405)	10.9	**0.001**
	STI symptom		11.4 (141/1234)	12.6	7.8 (110/1402)	8.4	0.056
	Sexually Active		50.0 (619/1239)	48.5	31.8 (447/1406)	31.9	**0.001**
Self-report sexually active (*N* = 1066)			*N* = 619		*N* = 447		
	Median Age		17 (IQR 16–19)		17 (IQR 16–19)		
	Age Range		12–28 years		13–23 years		–
	Age	< 15	15.2 (94/619)	17.4	18.8 (84/447)	19.5	0.612
		16–17	36.8 (228/619)	38.6	42.1 (188/447)	43.5	0.235
		18–19	36.7 (227/619)	34.0	25.7 (115/447)	24.5	**0.049**
		> 20	11.3 (70/619)	10.0	13.4 (60/447)	12.5	0.518
	HIV Prev		1.6 (10/619)	1.8	11.4 (51/447)	10.5	**< 0.001**
	HSV-2 Prev		3.9 (24/619)	3.6	21.0 (94/447)	21.0	**< 0.001**
	Pregnancy		Not applicable	–	6.3 (28/444)	6.2	Not applicable
	Prev Reported Preg/made pregnant^b^		49.3 (36/73)	52.4	95.7 (134/140)	95.0	**< 0.001**
	STI symptom		14.5 (89/614)	17.3	13.5 (60/444)	14.4	0.455
Self-report not sexually active (*N* = 1579)			*N* = 620		*N* = 959		
	Median Age		16 (IQR 15–17)		15 (IQR: 15–16)		
	Age Range		13–25 years		13–24 years		
	Age	< 15	32.1 (199/620)	32.8	52.2 (501/959)	51.9	**0.001**
		16–17	44.2 (274/620)	43.1	35.8 (343/959)	35.3	0.076
		18–19	18.2 (113/620)	18.0	9.6 (92/959)	9.9	**0.021**
		> 20	5.5 (34/620)	6.1	2.4 (23/959)	2.9	0.274
	HIV Prev		1.3 (8/620)	1.3	3.9 (37/959)	4.2	**0.001**
	HSV-2 Prev		2.3 (14/620)	1.9	5.6 (54/959)	5.7	**0.001**
	Pregnancy		Not applicable		2.2 (21/951)	2.5	Not applicable
	Prev Reported Preg/made pregnant		0.3 (2/613)	0.5	1.4 (13/952)	1.9	0.068
	STI symptom		8.5 (52/615)	9.3	5.3 (50/950)	6.0	0.125

Significant *p*-values ( ≤ 0.05) are set in bold

^a^Adjusted measures were calculated based on the cluster (school)-level summaries appropriate for school-based sampling

^b^ Percentage was calculated with regard to available data. Should be interpreted with caution as the denominator changed to 73 and 140 for male and females that self-reported as sexually active

As reported in Table 1, young women had a significantly higher prevalence in all the key MSAP variables than young men, other than the experience of STI symptoms where young men experienced a higher self-reported prevalence than women.

When we looked at the perceived risk of getting infected with HIV as a proxy for perceived risk of a negative health outcome, those participants reporting to be sexually active were significantly more likely to think that they were at higher risk (*p* = 0.025), whilst those who reported as not sexually active were more likely to report being at no risk of future HIV risk (*p* = 0.001) (Table 2).

**Table 2:** Risk perception of individuals by sexual activity in rural KwaZulu-Natal, South Africa (*N* = 223)

	Variable	Report Sexually Active unAdj % (*N* = 1066)	Adj %	Report Not sexually active unAdj % (*N* = 1579)	Adj %	*p*-value
Risk Perception		*N* = 899		*N* = 1433		
	No	204 (22.7)	22.9	507 (35.4)	33.7	**< 0.001**
	Low	191 (21.2)	19.5	244 (17.0)	17.1	0.174
	Some	87 (9.7)	8.6	136 (9.5)	9.1	0.723
	High	417 (46.4)	49.1	546 (38.1)	40.1	**0.025**

Significant *p*-values ( ≤ 0.05) are set in bold

### Prevalence of key MSAP variables amongst students reporting to be sexually active

Overall, more male students reported having had some type of sex before (p = 0.001), within those self-reporting as sexually active more males reported having been sexually active (58.1%) than females (41.9%). The overall prevalence of key variables in those reporting to be sexually active was, HIV prevalence 5.7%, HSV-2 prevalence 11.1%, positive pregnancy test 6.3%, self–reported ever made someone pregnant or previous pregnancy 79.8% and self-reported STI symptoms 14%. The gender difference in the prevalence of MSAP variables persisted, consistent with the gender disparity seen nationally and regionally [6]. HIV prevalence amongst sexually active young women was approximately seven times greater than their male counterparts, with young women’s prevalence 11.4% to the males 1.6% (*p* < 0.001). HSV-2 prevalence amongst self-reporting sexually active women was significantly higher compared to young men, reporting a prevalence of 21.0% compared to 3.9% in young men (*p* < 0.001). Amongst the key self-reported behavioural markers of sexual activity, the gender disparity continued, significantly more females reported having had a previous pregnancy, compared to young men who made someone pregnant before (*p* < 0.001). Previous pregnancy prevalence for those reporting sexually active should be interpreted cautiously as the available data had high missing values. For those reporting symptoms of an STI, the gender disparity was not significantly different, 13.5% of females reporting to have been sexually active had a symptom of an STI as opposed to 14.5% of young men self-reporting STI symptoms. For young women, it appeared that they were engaging in unprotected sex, indicated by the high pregnancy prevalence (6.3%).

### Prevalence of key MSAP variables amongst students reporting to not be sexually active

Overall, fewer young men reported that they had never had sex before (*p* = 0.001). For those self-reporting never having engaged in any type of sex before, fewer males (39.3%) reported not having had sex before compared to females (60.7%). For those reporting to have never been sexually active, the overall prevalence of MSAP variables were HIV prevalence 2.8%, HSV-2 prevalence 4.3%, positive on pregnancy test 2.2%, ever having made someone pregnant or previous pregnancy prevalence was 1% and positive for a self-reported STI symptom was 6.5%. The gender disparity amongst the prevalence of the key indicators of sexual activity remained skewed towards females (Fig. 1) in almost all the variables. HIV prevalence amongst females reporting to have never been sexually active was significantly higher (3.9%) than amongst males (1.3%) (*p* = 0.001). Young women had four times higher HIV prevalence when compared to their male counterparts. Amongst those reporting not being sexually active the difference in HSV-2 prevalence was less pronounced between the genders but still significantly different. In females, the prevalence of HSV-2 was 5.6% compared to 2.3% in males (*p* = 0.001), with females having two times greater prevalence than males. Evidence of unprotected sexual encounters was again evident amongst young females reporting no sexual activity where there was a 2.2% prevalence of pregnancy. For the key self-reported behavioural markers that could indicate sexual activity, there was no significant difference between the genders. Firstly, the prevalence of ever been/ever made someone pregnant (1.2% of females and 0.5% of males (*p* = 0.068)), suggest possible evidence of unsafe sexual practices, despite reporting to have never had sex. Secondly, amongst those reporting never having had sex, 5.3% of females reported to having a positive STI screening symptom. Here the gender disparity was reversed, young men had more reported positive STI screening symptoms than females, with 9.3% of young men experiencing at least one STI symptom by self-report, however, the difference was not significant (*p* = 0.125).

### Differences between reported sexual activity and newly identified as sexually active

In total, 59.7% of those students that enrolled self-reported as having never had sex before. We identified 14.1% of these students who appeared to have been sexually active because of the presence of at least one sexual health outcome. Of the 223 sexually active individuals newly identified using the MSAP variables, female students were more likely to under-report sexual activity (*p* < 0.001) (Table 3). In the 223 individuals, 20.2% were identified by looking at HIV in those reporting to as not sexually active, an additional 27.8% were identified by adding HSV-2, 6.7% were added by adding pregnancy results, 3.6% were added by adding ever pregnant/ever made someone pregnant and the final 41.7% were added by adding self-reported STI symptom. Some variables overlapped, and the breakdown of the variables for those newly identified sexually active individuals are presented in Table 3.

**Table 3:** Descriptive breakdown of the multicomponent risk profile for those students identified as newly sexually active (*N* = 223)

Multi-component Risk Profile	Newly identified sexually active male (n/*N* = 75)		Adj. %	Newly identified sexually active female (n/*N* = 148)	Adj. %	*p*-value
Overall newly identified	33.6 (75/223)		31.97	66.4 (148/223)	68.0	< 0.001
Multi-component Risk Profile	Variable		Overall % N = 223	Male % N = 75	Female % N = 148	
Age of students newly identified as sexually active	Median Age		16 years (IQR: 15–18)	16 years (IQR 15–16)	16 (IQR 16–17)	
	Age Range		13–24 years	14–23 years	13–24 years	
		< 15	35.9 (80/223)	26.7 (20/75)	40.5 (60/148)	
		16–17	36.3 (81/223)	40.0 (30/75)	34.5 (51/148)	
		18–19	20.2 (45/223)	24.0 (18/75)	18.2 (27/148)	
		> 20	7.6 (17/223)	9.3 (7/75)	6.8 (10/148)	
Multi-component Risk Profile						
Only self-reported STI symptom			41.7% (93)	69% (51/74)	28% (42)	
Only HIV			14.3% (32)	11% (8)	16% (24)	
Only HSV-2			22.9% (51)	18% (13)	26% (38)	
Only positive on pregnancy test			6.7% (15)	n/a	10% (15/147)	
Only ever made someone pregnant/previous pregnancy			3.1% (7)	3% (2/74)	3.4% (5)	
HIV*STI symptom			0.9% (2)	0% (0)	1.4% (2)	
Positive on pregnancy test*HIV			0.4% (1)	0% (0)	0.7% (1)	
Ever made someone pregnant/previous pregnancy*STI			0.4% (1)	0% (0)	0.7% (1)	
Ever made someone pregnant/previous pregnancy*HIV			1.8% (4)	0% (0)	3% (4)	
HSV2*STI symptom			2.2% (5)	1.3% (1)	3% (4)	
HIV*HSV-2			2.2% (5)	0% (0)	3.4% (5)	
Positive on pregnancy test*HSV-2			1.3% (3)	n/a	2% (3)	
Ever made someone pregnant/previous pregnancy*HSV-2			0.9% (2)	0% (0)	1.4% (2)	
HSV-2* ever made someone pregnant/previous pregnancy*Preg test			0.4% (1)	n/a	0.7% (1)	
HSV-2*Preg*HIV*STI symptom			0.4% (1)	n/a	0.7% (1)	

In total, MSAP increased the number identified as sexually active from 40.3 to 48.7%. The basic age demographic for the 223 newly identified sexually active students is presented in Table 3.

Characterising those newly identified as sexually-active compared to those who self-reported as sexually active on various ecological level factors highlighted some important differences (Table 4 in the appendix section). At an individual level, those newly-identified as sexually active were more likely to be female (*p* = < 0.000), 15 years old or younger (*p* = 0.008), 20-year-old or older (0.040), less likely to perceive being at risk (*p* = 0.037) or have ever used alcohol (*p* = < 0.000). At a relational level, newly-identified as sexually active were less likely to report having ever had a boyfriend/girlfriend (*p* = 0.000) or ever having had more than one boyfriend/girlfriend at the same time (*p* = 0.000). They were also less likely to have felt pressured to have sex by their peers (*p* = < 0.000) or partners (*p* = 0.008), or have friends that were mostly boys (*p* = 0.000). At a familial level, those newly identified were more likely to be of medium socioeconomic (SES) status (p = 0.037) and less likely to be low SES (*p* = 0.029). At a school level, those newly-identified as sexually active were less likely to have repeated a grade (*p* = 0.024) and were more likely to be engaged in social activities (*p* = 0.032) at a community level.

**Table 4:** Ecological level characteristics of newly identified sexually active individuals compared to those who self-reported sexually active in rural KwaZulu-Natal, South Africa

L^a^	Variables	Categories	Reported Sexually Active (N = 1066)			Newly identified as sexually active (N = 223)			
			n/N	Unadjusted	Adjusted %	n/N	Unadjusted	Adjusted %	*p*-value
I	Biological Sex	Male	619/1066	58.1	57.6	75/223	33.6	32.0	**< 0.000**
		Female	447/1066	41.9	42.4	148/223	66.4	68.0	**< 0.000**
I	Age	≤15 years old	178/1066	16.70	18.0	80/223	35.9	35.2	**0.008**
		16–17 years old	416/1066	39.02	40.6	81/223	36.3	35.2	0.302
		18–19 years old	342/1066	32.08	30.1	45/223	20.2	19.9	**0.040**
		≥20 years old	130/1066	12.20	11.3	17/223	7.6	9.6	0.757
I	Importance of going to school	Not Important	29/1050	2.8	2.6	5/221	2.3	2.3	0.719
		Important	1021/1050	97.2	97.4	216/221	97.7	97.7	0.719
I	Importance of not falling pregnant at school	Not Important	299/1033	28.9	28.5	62/221	28.1	28.9	0.918
		Important	734/1033	71.1	71.5	159/221	71.9	71.1	0.920
I	Currently using contraception	No	241/465	51.8	53.2	19/37	48.6	45.6	0.398
		Yes	224/465	48.2	46.5	18/37	51.4	54.4	0.523
I	Risk Perception	No risk	204/899	22.7	22.9	66/199	33.2	32.4	**0.036**
		At risk	695/899	77.3	77.1	133/199	66.8	67.6	**0.037**
I	Alcohol Use	No	665/1036	64.2	64.2	181/220	17.7	83.1	**< 0.000**
		Yes	371/1036	35.8	35.8	39/220	82.3	16.9	**< 0.000**
I	Ever HIV test	No	588/1044	56.3	57.1	132/220	60.0	59.0	0.764
		Yes	456/1044	43.7	42.9	88/220	40.0	41.0	0.764
R	Ever had a boyfriend/girlfriend	No	223/1052	21.2	21.1	97/223	43.5	42.6	**0.000**
		Yes	829/1052	78.8	78.9	126/223	56.5	57.4	**0.000**
R	Ever more than one boyfriend/girlfriend at the same time	No	458/903	50.7	51.2	97/138	70.3	71.2	**0.000**
		Yes	445/903	49.3	48.8	41/138	29.7	28.8	**0.000**
R	Pressure to have sex (peers)	No	677/1064	63.6	63.5	199/222	89.6	89.1	**< 0.000**
		Yes	387/1064	36.4	36.5	23/222	10.4	10.9	**< 0.000**
R	Pressure to have sex (partner)	No	708/1061	66.7	66.6	112/115	97.4	91.3	**0.003**
		Yes	353/1061	33.3	33.4	3/115	2.6	8.7	**0.008**
R	Gender of friends	Both boys and girls	217/1008	21.5	22.2	39/214	18.2	17.7	0.141
		Mostly girls	351/1008	34.8	35.5	119/214	55.6	57.1	**0.000**
		Mostly boys	440/1008	43.7	42.3	56/214	26.2	25.3	**0.000**
F	Head of Household	Both parents	146/1062	13.7	14.4	41/221	18.6	19.4	0.141
		Birth Mother	371/1062	34.9	34.7	68/221	30.8	30.3	0.254
		Birth Father	163/1062	15.3	15.0	33/221	14.9	15.0	0.989
		Child headed	22/1062	2.1	2.0	3/221	1.4	1.3	0.481
		Grandparent	182/1062	17.1	17.3	43/221	19.5	19.0	0.607
		Sibling > 18	29/1062	2.7	2.9	4/221	1.8	2.0	0.413
		Other	149/1062	14.0	13.8	29/221	13.1	13.0	0.696
F	Social grant	None	142/1060	13.4	13.1	32/223	14.3	15.1	0.490
		One	488/1060	46.0	46.5	110/223	49.3	47.9	0.699
		> 1	318/1060	30.0	30.5	60/223	26.9	27.9	0.441
		Do not know	112/1060	10.6	10.0	21/223	9.4	9.2	0.759
F	Socio-economic Status	High	411/1064	38.6	40.5	89/223	39.9	40.6	0.997
		Medium	413/1064	38.8	37.6	101/223	45.3	45.4	**0.037**
		Low	240/1064	22.6	21.9	33/223	14.8	14.0	**0.029**
F	No adult deaths	None	525/1052	49.9	49.8	116/222	52.3	52.5	0.411
		One	202/1052	19.2	20.2	41/222	18.5	17.5	0.450
		More than One	325/1052	30.9	30.0	65/222	29.3	30.0	0.980
S	Repeated a grade	No	405/1055	38.4	39.1	128/218	58.7	57.4	**0.023**
		Yes	650/1055	61.6	60.9	90/218	41.3	42.6	**0.024**
S	Absenteeism	0 days	154/1002	15.4	16.3	39/212	18.4	18.4	0.529
		1–4 days	547/1002	54.6	55.4	123/212	58.0	57.3	0.670
		5–10 days	181/1002	18.1	17.4	30/212	14.2	15.3	0.608
		> 10 days	120/1002	12.0	10.9	20/212	9.4	8.9	0.425
C	Social Participation	No	83/1063	7.8	7.7	10/223	4.5	4.1	**0.033**
		Yes	980/1063	92.2	92.3	213/223	95.5	96.0	**0.032**

^a^Refers to ecological levels: *I* individual, *R* relational, *F* family, *S* school, *C* Community

Significant *p*-values ( ≤ 0.05) are set in bold

To further understand whether those newly identified as sexually active perceived that they were at risk of negative health outcomes we used their self-reported perception of future HIV infection as a proxy marker. For those reporting that they felt they were at ‘some risk’ of future HIV infection, significantly more male students reported to feel at some risk than female students (Table 5). This subset of students, while being part of a group that perceived themselves to be a lower risk (Table 4), appeared to correctly assess that they may still be at some risk. When assessing the risk perception between those who experienced a negative health outcome in the self-reported sexually active and newly identified as sexually active (Table 6), the data showed that amongst those newly identified, those with HSV-2, those who had been pregnant before or those who self-reported an STI symptom were more likely to perceive themselves as being at lower risk.

**Table 5:** Risk perception of newly identified sexually active individuals in rural KwaZulu-Natal, South Africa (*N* = 223)

Sample Group	Variable	Overall % N = 223	Adj. %	Male % *N* = 75	Adj. %	Female % *N* = 148	Adj. %	Adj. p
Risk Perception	No Risk	33.2 (66/199)	32,4	28.6 (18/63)	31,9	35.3 (48/136)	33,9	0.838
	Low Risk	16.6 (33/199)	16,9	19.0 (12/63)	17,3	15.4 (21/136)	15,8	0.818
	Some Risk	10.6 (21/199)	10,6	15.9 (10/63)	15,3	8.1 (11/136)	8,5	**0.002**
	High Risk	39.7 (79/199)	40,1	36.5 (23/63)	35,4	41.2 (56/136)	41,9	0.522

Significant *p*-values ( ≤ 0.05) are set in bold

**Table 6:** Risk perception of highest risk self-report sexually active compared to the newly identified sexually active individuals in rural KwaZulu-Natal, South Africa

Sample Group	Variable	Self-reported sexually active		Newly identified as sexually active		Adj. p
Risk Perception		HIV+ (n = 61)	Adj %	HIV+ (*n* = 45)	Adj %	
	No risk	14.8% (8/54)	10.2	26.8% (11/41)	25.85	0.113
	At risk	54.8% (46/84)	89.8	73.2%(30/41)	74.15	0.113
		HSV+ (*n* = 118)	Adj %	HSV+ (*n* = 68)	Adj %	
	No risk	16.0% (17/106)	16.4	31.7% (19/60)	32.3	0.088
	At risk	84.0% (89/106)	76.4	68.3% (41/60)	67.7	0.417
		Preg + (*n* = 28)	Adj %	Preg + (*n* = 21)	Adj %	
	No risk	21.7% (5/23)	21.4	30% (6/20)	25	0.787
	At risk	78.3% (18/23)	64.3	70% (14/20)	67.8571	0.82
		Ever Preg (*n* = 170)	Adj %	Ever Preg (*n* = 15)	Adj %	
	No risk	24.2% (36/149)	24.0	25% (3/12)	7.1	**0.016**
	At risk	75.8% (113/149)	76.0	75% (9/12)	42.9	**0.026**
		STI+ (*n* = 149)	Adj %	STI+ (*n* = 102)	Adj %	
	No risk	15.6% (19/122)	14.2	37.1% (33/89)	39.4	**0.002**
	At risk	84.4% (103/122)	85.8	62.9% (56/89)	60.6	**0.002**

Significant *p*-values ( ≤ 0.05) are set in bold

## Discussion

Using the MSAP approach we identified more sexually active high-school students than we would have using only a self-reported measure. Our findings are consistent with previous research which suggests a trend in under-reporting sexual activity amongst young people [2, 4, 13]. This highlights that the continued reliance on only self-reported measures is susceptible underestimating the proportion of sexually active adolescents [2, 4]. Considering that sexual activity is highly related to increased risk of negative health outcomes [1, 9, 34–36], using MSAP as a basis to understand the predictors of sexual health outcomes, and then prevent future negative health outcomes is important.

Separating by MSAP variables, HIV positive status identified an additional 1.7% of students who should have been included as sexually active, HSV-2 positive status identified an additional 2.3%, while positive pregnancy tests identified an additional 0.6%. Using self-report variables, ever pregnant/made someone pregnant identified an additional 0.3%, and self-report STI symptoms an additional 3.5% of students who should have been included as sexually active. Indicative of high risk, 11.2% of the newly identified students were positive on multiple MSAP variables. In addition to identifying a larger proportion of sexually active students, MSAP identified high-risk students experiencing negative health outcomes that increase their risk of future HIV infection.

Previous research suggests particular difficulty in identifying sexually active young women [2, 12]. Using MSAP variables we were able to identify an additional 223 potentially sexually active students, and almost double the number of those newly identified were young women. The disparity between young women and men reporting sexual activity could be a result of social desirability, female vs male gender norm expectations or how young people understand the term sexually active [2, 3, 13, 21]. Failing to identify a proportion of sexually active young people (in particular young women) is problematic considering the association between sexual activity and the risk of HIV, STI and early pregnancy.

The MSAP approach highlighted the importance of STIs, HSV-2, pregnancy and HIV as indicators of unprotected sex [8, 10, 34]. Reducing HIV endpoints remains, understandably, the most important outcome for most research studies. However, low seroconversion rates in adolescent and young cohorts means assessing the efficacy of prevention trials can be difficult [35, 37]. One problem with focusing only on HIV as an endpoint is failing to recognise the importance of young people who are sexually active, having high risk, and unprotected sexual encounters but are not yet infected with HIV. In the 223 newly identified sexually active individuals, using only HIV endpoints to define engagement in unprotected sexual activity, we would have excluded 80% of those whose risk profile suggested high-risk sexual encounters and a future risk of HIV infection. Using a MSAP approach we can create nuanced risk categories, identifying those who are sexually active and 1) already experiencing negative health outcomes 2) are HIV negative but experiencing negative health outcomes that put them at high risk of future seroconversion, and 3) those who are sexually active with no negative health outcomes, in addition to those who are not sexually active. This highlights the huge potential for identifying these particularly at-risk students and focusing HIV prevention efforts.

Comparing those who reported being sexually-active with those newly-identified raised some interesting insights into those adolescents who do not disclose sexual activity. When looking at age-stratifications, the findings suggested that the willingness to disclose sexual activity may change over time. Amongst all those students included, a higher proportion of those that self-disclosed sexual activity were older than 16, and male. Amongst the 223 newly identified sexually active students, this trend was observed again, the majority of these students were under 17 years old. This finding is congruent with other studies which have shown that young women 15–19 years were less willing to report marriages and first births before age 15 than were women from the same group when asked again five years later [12]. This unwillingness to report sexual activity is likely due to broader cultural and social factors which govern the acceptability of sexual activity amongst students [11, 12, 38].

Other differences between those identified as newly sexually-active raise some interesting hypotheses. The relational, familial, school and community level descriptions suggest that those who do not self-report sexually-active may be those who perceive negative consequences of admitting sexual activity. Besides being more likely to be female, a higher proportion of newly-identified students appear to successfully manage school, come from higher-income households, and participate in extramural activities, less likely to drink alcohol or admit relationships and feel less pressure to have sex from peers or partners. It appears the newly-identified students may be those that either fall outside what people usually define as vulnerable or at-risk. It is possible that for HIV infection vertical transmission may be the cause of infection for some adolescents but for the other MSAP variables, sexual activity is the most likely route of infection. Therefore, the newly-identified may be young people who, 1) come from households where being sexually-active could have negative social implications, 2) come from social groups where being sexually-active is less acceptable, 3) may be young people that choose to be sexually active but are unwilling to admit it because of perceived social or personal repercussions, or 4) come from households/social groups with more conservative values. These factors could make this sub-set of adolescents particularly sensitive to possible confidentiality breaches by peers, school staff, health-care workers, researchers or other social connections [1]. This may have implications for health-seeking behaviour within this group because this group is most likely less inclined to seek out health-care services that require disclosure of sexual activity. The MSAP approach could provide an innovative conceptualisation for identifying a larger proportion of these younger sexually-active individuals.

While MSAP identified a greater proportion of sexually active students, it was also able to identify a particularly at-risk group who experienced at least one negative health outcome. The presence of these negative health outcomes are important when thinking about future HIV risk [29]. Using MSAP variables we noted a disconnect between risk perception and actual risk when investigating the difference in risk perception between those who were sexually active and had a negative health outcome and those who reported being not sexually active but had a negative health outcome. Those who self-reported as not sexually active and had HSV-2 infection, self-reported previous pregnant/made someone pregnant or having an STI symptom were more likely to report that they were at no risk of HIV infection when compared to the same group who reported to be sexually active. This suggests that those students reporting as not sexually active may be reporting socially desirable responses, trust their partners if they have them, or have optimism bias, believing that negative outcomes will not happen to them, underestimating their perceived risk in comparison to their real risk [29]. It suggests that students may not treat all negative health outcomes with equal importance, not realising that other high-risk outcomes (pregnancy/HSV-2/STI infection) increase future risk of HIV. Considering 79.8% of newly identified students had experienced a negative health outcome that increased their risk of HIV but were HIV negative, using MSAP has great potential for building contextualised understandings of risk perception and targeting them with prevention interventions.

Considering the need to include adolescents, students and young people in HIV prevention and clinical trials [39, 40] being able to identify at-risk youth is imperative for the field. Most studies and pre-screening protocols rely on questions such as “have you had sex in the last 30 days?” or “have you ever had sex?” to identify at-risk populations. Using MSAP in addition to innovative methodologies such as cognitive interviewing [41, 42] during formative stages of study design may assist in constructing more reliable tools, and allow us to improve how we measure health outcomes. Creating pre-screening protocols that assess multiple variables associated with sexual activity, as shown in this analysis, provides a strong theoretical and medically-informed profile for assessing sexual activity and increased risk as well as linking HIV positive young people to treatment.

MSAP provides a methodological and theoretical platform from which to improve how we define sexual activity and risk profiles in order to improve our interpretation of risk amongst young people. A key limitation of the current study is that we had high missing data on certain variables, in particular the self-reported variable of ever pregnant/ever made someone pregnant. Therefore further investigation is required regarding these data, and results should be interpreted with caution on this variable. Additonally, self-reported measures may under-estimate or over-estimate the number of potentially sexually active students. In particular, and that there may have been misreporting on some of the behavioural variables and therefore future work on a MSAP approach is required. We note that the inclusion of self-reported STI symptoms increased the number of students we identified as potentially sexually active. The main study included two male students over 24 years of age, but both already reported being sexually active, not affecting the MSAP profile. However, future work investigating the usefulness of MSAP in identifying sexual active individuals in different age groups is required. The design of the parent study (a cluster randomised control trial, limited the analyses for the current study, highlighting the need for future work. This study also took place with high school students in rural KwaZulu-Natal, so future work in other populations in required and generalisability to other settings may be limited.

Further research to refine the components of the profile and differences between those that self-identify as sexually-active and those identified using MSAP variables is required. The next steps for this research will include looking at how the MSAP informed risk profiles (sexually active with negative health outcome/sexually active and no negative health outcome) are linked to increased risk of negative health outcomes and how these change across time.

## Conclusion

We know that high-school students are sexually active and are having sexual encounters that put them at risk of negative sexual health outcomes. Our analysis suggests the current methods of assessing sexual activity fail to identify an important portion of sexually-active students. If further research suggests that using and adapting the MSAP variables will assist in identifying a greater proportion of sexually active youth, then it could become a useful tool for identifying adolescents for inclusion in research, and characterising adolescents who avoid disclosing sexual-activity. Future research into the predictors of MSAP profiles could strengthen its use as a prevention tool and aid in the development of screener tools that could be used in the public health care and research settings. The MSAP provides a framework to design more nuanced analyses of risk in sexually active young people, comparing those who are seemingly more resilient, not experiencing negative sexual health outcomes with those that have despite exposure to similar settings of risk. Using an MSAP approach provides a novel approach to defining actual sexual activity in adolescents and a platform for improving our understanding of adolescent sexual health outcomes.

## Abbreviations

CAPRISA: Centre for the AIDS Programme of research in South Africa.
HIV: Human Immunodeficiency Virus
HSV-2: Herpes Simplex-2 Virus
MSAP: Multi-component sexual activity profile
SRH: Sexual reproductive health
SSA: Sub-Saharan Africa
STI: Sexually Transmitted Infection

## References

[1] Patton GC, Sawyer SM, Santelli JS, Ross DA, Afifi R, Allen NB (2016). Our future: a lancet commission on adolescent health and wellbeing. Lancet.

[2] Darroch JE, Singh S, Woog V, Bankole A, Ashford LS. Research gaps in adolescent sexual and reproductive health. 2016; Guttmacher Institute. 1–7. Available at https://www.guttmacher.org/report/research-gaps-in-sexual-and-reproductive-health. Accessed 18 Mar 2018.

[3] Hindin MJ, Christiansen CS, Ferguson BJ (2013). Setting research priorities for adolescent sexual and reproductive health in low- and middle-income countries. Bull World Health Organ.

[4] Michielsen K, Chersich MF, Luchters S, De Koker P, Van Rossem R, Temmerman M (2010). Effectiveness of HIV prevention for youth in sub-Saharan Africa: systematic review and meta-analysis of randomized and nonrandomized trials. AIDS.

[5] UNFPA (2016). The State of World Population 2016.

[6] UNAIDS. Gap Rep 2016. Available at http://www.unaids.org/sites/default/files/media_asset/2016-prevention-gap-report_en.pdf

[7] Looker KJ, Garnett GP, Schmid GP (2008). An estimate of the global prevalence and incidence of herpes simplex virus type 2 infection. Bull World Health Organ.

[8] Kharsany ABM, Mlotshwa M, J a F, Yende Zuma N, Samsunder N, Abdool Karim SS (2012). HIV prevalence among high school learners - opportunities for schools-based HIV testing programmes and sexual reproductive health services. BMC Public Health.

[9] Shisana, O, Rehle, T, Simbayi LC, Zuma, K, Jooste, S, Zungu N, Labadarios, D, Onoya D et al. South African national HIV prevalence, incidence and behaviour survey, 2012. Human Science Research Council. 10.2989/16085906.2016.1153491 27002359

[10] Abdool Karim Q, Kharsany ABM, Leask K, Ntombela F, Humphries H, Frohlich JA (2014). Prevalence of HIV, HSV-2 and pregnancy among high school students in rural KwaZulu-Natal, South Africa: a bio-behavioural cross-sectional survey. Sex Transm Infect.

[11] Mmari K, Blum RW (2009). Risk and protective factors that affect adolescent reproductive health in developing countries: a structured literature review. Glob Public Health.

[12] Neal SE, Hosegood V (2015). How reliable are reports of early adolescent reproductive and sexual health events in demographic and health surveys?. Int Perspect Sex Reprod Health.

[13] Hindin MJ, Truncalp O, Gerdts C, Gipson JD, Say L (2016). Monitoring adolescent sexual and reproductive health. Bull World Health Organ.

[14] Sedgh G, Finer LB, Bankole A, Eilers MA, Singh S (2015). Adolescent pregnancy, birth, and abortion rates across countries: levels and recent trends. J Adolesc Health.

[15] Bearinger LH, Sieving RE, Ferguson J, Sharma V (2007). Global perspectives on the sexual and reproductive health of adolescents: patterns, prevention, and potential. Lancet.

[16] Padian NS, Mccoy SI, Karim SSA, Hasen N, Kim J, Bartos M, et al. Review HIV prevention transformed: the new prevention research agenda. Lancet. 2011;378:269–78. 10.1016/S0140-6736(11)60877-5 PMC3606928 21763938

[17] Sawyer SM, R a A, Bearinger LH, Blakemore S-J, Dick B, Ezeh AC (2012). Adolescence: a foundation for future health. Lancet.

[18] Patton GC, Coffey C, Cappa C, Currie D, Riley L, Gore F (2012). Health of the world’s adolescents: a synthesis of internationally comparable data. Lancet..

[19] Viner RM, Hargreaves DS, Ward J, Bonell C, Mokdad AH, Patton G (2017). The health benefits of secondary education in adolescents and young adults: an international analysis in 186 low-, middle- and high-income countries from 1990 to 2013. SSM - Popul Heal.

[20] de Oliveira T, Kharsany ABM, Gräf T, Cawood C, Khanyile D, Grobler A (2016). Transmission networks and risk of HIV infection in KwaZulu-Natal, South Africa: a community-wide phylogenetic study. Lancet HIV.

[21] Blum RW, Bastos FIPM, Kabiru CW, Le LC (2012). Adolescent health in the 21st century. Lancet..

[22] Magnani R, Ph D, Macintyre K, Karim AM, Brown L, Hutchinson P (2005). The impact of life skills education on adolescent sexual risk behaviors in KwaZulu-Natal, South Africa. J Adolesc Health.

[23] Cluver L, Boyes M, Orkin M, Pantelic M, Molwena T, Sherr L (2013). Child-focused state cash transfers and adolescent risk of HIV infection in South Africa: a propensity-score-matched case-control study. Lancet Glob Heal.

[24] Cluver L, Boyes M, Orkin M, Sherr L (2013). Poverty, AIDS and child health: identifying highest-risk children in South Africa. South African Med J.

[25] Christofides NJ, Jewkes RK, Dunkle KL, McCarty F, Jama Shai N, Nduna M (2014). Risk factors for unplanned and unwanted teenage pregnancies occurring over two years of follow-up among a cohort of young south African women. Glob Health Action.

[26] Harrison A, Colvin CJ, Kuo C, Swartz A, Lurie M (2015). Sustained high HIV incidence in young women in southern Africa: social, behavioral, and structural factors and emerging intervention approaches. Curr HIV/AIDS Rep.

[27] Pettifor A, Bekker L-G, Hosek S, DiClemente R, Rosenberg M, Bull SS (2013). Preventing HIV among young people: research priorities for the future. J Acquir Immune Defic Syndr.

[28] SANAC. National Strategic Plan on HIV, STIs and TB 2012-2016. Sanac. 2011;84:1-78.

[29] Macintyre K, Rutenberg N, Brown L, Karim A (2004). Understanding perceptions of HIV risk among adolescents in KwaZulu-Natal. AIDS Behav.

[30] Abdool Karim Q, Leask K, Kharsany A, Humphries H, Ntombela F, Samsunder N (2015). Impact of conditional cash incentives on HSV-2 and HIV prevention in rural south African high school students: results of the CAPRISA 007 cluster randomized controlled trial. J Int AIDS Soc.

[31] Kirby D. Antecedents of adolescent initiation of sex, contraceptive use, and pregnancy. Am J Health Behav. 26:473–85. 10.5993/ajhb.26.6.8 12437022

[32] Bronfenbrenner U. Ecological models of human development. In International Encyclopedia of Education, Volume 3, 2nd Edition. Oxford: Elsevier; 1994. Reprinted in Gauvain, M and Cole, M (Eds.), Readings in the Development of Children, 2nd Edition. NY: Freeman; 1997. p. 37–43.

[33] Crowder M (2009). Cluster randomised trials by Richard J. Hayes, Lawrence H. Moulton. Int Stat Rev.

[34] Dellar RC, Dlamini S, Karim QA (2015). Adolescent girls and young women: key populations for HIV epidemic control. J Int AIDS Soc.

[35] Dellar R, Waxman A, Karim QA (2015). Understanding and responding to HIV risk in young south African women: clinical perspectives. South African Med J.

[36] Department of Health E and SD (2013). The 2013 National Antenatal Sentinel HIV prevalence survey South Africa.

[37] Pettifor A, MacPhail C, Selin A, Gómez-Olivé FX, Rosenberg M, Wagner RG (2016). HPTN 068: a randomized control trial of a conditional cash transfer to reduce HIV infection in young women in South Africa—study design and baseline results. AIDS Behav.

[38] Kotchick BA, Shaffer A, Forehand R, Miller KS (2001). Adolescent sexual risk behavior: a multi-system perspective. Clin Psychol Rev [Internet].

[39] DiClemente RJ, Ruiz MS, Sales JM (2010). Barriers to adolescents’ participation in HIV biomedical prevention research. J Acquir Immune Defic Syndr.

[40] Nelson RM, Lewis LL, Struble K, Wood SF (2010). Ethical and regulatory considerations for the inclusion of adolescents in HIV biomedical prevention research. J Acquir Immune Defic Syndr.

[41] Lee J (2014). Conducting cognitive interviews in cross-National Settings. Assessment.

[42] Willis GB (2005). Cognitive interviewing: a tool for improving questionnaire design.

